# Specific detection and differentiation of classic goose parvovirus and novel goose parvovirus by TaqMan real-time PCR assay, coupled with host specificity

**DOI:** 10.1186/s12917-019-2090-7

**Published:** 2019-11-01

**Authors:** Chunhe Wan, Cuiteng Chen, Longfei Cheng, Rongchang Liu, Shaohua Shi, Guanghua Fu, Hongmei Chen, Qiuling Fu, Yu Huang

**Affiliations:** 1 Institute of Animal Husbandry and Veterinary Medicine of Fujian Academy of Agricultural Sciences, Fuzhou, 350013 China; 2 Fujian Provincial Key Laboratory for Avian Diseases Control and Prevention & Fujian Animal Diseases Control Technology Development Center, Fuzhou, 350013 China

**Keywords:** Classic GPV, Differentiation, N-GPV, NS gene, Specific detection, TaqMan real-time PCR assay

## Abstract

**Background:**

Classic goose parvovirus (cGPV) causes high mortality and morbidity in goslings and Muscovy ducklings. Novel GPV (N-GPV) causes short beak and dwarfism syndrome (SBDS) in Cherry Valley ducks, Pekin ducks and Mule ducks. Both cGPV and N-GPV have relatively strict host specificity, with obvious differences in pathogenicity. Specific detection of cGPV and N-GPV may result in false positives due to high nucleotide similarity with Muscovy duck parvovirus (MDPV). The aim of this study was to develop a highly specific, sensitive, and reliable TaqMan real-time PCR (TaqMan qPCR) assay for facilitating the molecular detection of cGPV and N-GPV.

**Results:**

After genetic comparison, the specific conserved region (located on the NS gene) of cGPV and N-GPV was selected for primer and probe design. The selected regions were significantly different from MDPV. Through a series of optimization experiments, the limit of detection was 50.2 copies/μl. The assay was highly specific for the detection of cGPV and N-GPV and no cross-reactivity was observed with *E. coli., P.M., R.A.*, S.S., MDPV, N-MDPV, DAdV-A, DEV, GHPV, DHAV-1, DHAV-3, ATmV, AIV, MDRV and N-DRV. The assay was reproducible with an intra-assay and inter-assay variability of less than 2.37%. Combined with host specificity, the developed TaqMan qPCR can be used for cGPV and N-GPV in differential diagnoses. The frequency of cGPV in Muscovy duckling and goslings was determined to be 12 to 44%, while N-GPV frequency in Mule ducks and Cherry Valley ducks was 36 to 56%. Additionally, fluorescence-positive signals can be found in Mule duck embryos and newly hatched Mule ducklings. These findings provide evidence of possible vertical transmission of N-GPV from breeding Mule ducks to ducklings.

**Conclusions:**

We established a quantitative platform for epidemiological investigations and pathogenesis studies of cGPV and N-GPV DNA that was highly sensitive, specific, and reproducible. N-GPV and cGPV infections can be distinguished based on host specificity.

## Background

Waterfowl parvoviruses, including goose parvoviruses (GPVs), Muscovy duck parvoviruses (MDPVs) and the variant viruses of GPVs and MDPVs, were renamed as *Anseriform dependoparvovirus 1* by the International Committee on Taxonomy of Viruses (ICTV) and have been assigned to the genus *Dependoparvovirus* in subfamily Parvovirinae under family Parvoviridae based on similarities in phylogenetic properties (https://talk.ictvonline.org/taxonomy/). These viruses contain a linear, single-stranded DNA genome (approximately 5.1 kb in length). Both the 5′-terminal and 3′-terminal ends of these viruses have two inverted terminal repeats (ITR) forming a hairpin structure. There are two main open reading frames (ORFs). The left ORF encodes the non-structural protein (NS) responsible for both viral replication and regulation. The right ORF encodes the structural proteins VP1, VP2 and VP3. The VP2 and VP3 contain the same carboxyl-terminal portion as VP1 in these viruses [1–3].

GPV infection, also known as Derzsy’s disease in Europe, was described in China by Professor Fang in the early 1960s [4]. The virus mainly affects goslings and Muscovy ducklings that are less than one-month-old. Muscovy duck parvovirus infection, also known as “three-week” disease in China, was initially described by Professor Lin in our laboratory in the early 1990s [5]. In contrast to GPV, MDPV infection occurs only in Muscovy ducklings and is characterized by watery diarrhoea, wheezing, and locomotor dysfunction. Both GPVs and MDPVs infections are widespread in China, causing huge economic loss due to the high mortality and morbidity within waterfowl husbandry industries.

Genomic comparison of GPV (strain B, GenBank accession number U25749) and MDPV (strain FM, GenBank accession number U22967) [2] indicated 82.1% nucleotide similarity at the genome level. Furthermore, these strains shared 83.0 and 90.6% nucleotide and amino acids similarity at the NS level and 81.5 and 87.6% nucleotide and amino acids at the VP1 level, respectively. The high similarity at the nucleotide and amino acids level between GPVs and MDPVs may cause false positive results due to MDPV contamination when using a GPV-specific diagnosis method for Muscovy ducklings.

Real-time PCR is an extremely useful tool that has been widely used for viral diagnostic applications. The TaqMan probe, which was designed to bind to a specific region of the target DNA, has shown improved specificity when distinguishing between closely related strains with high nucleotide similarity [6–8]. The TaqMan-based real-time PCR method (TaqMan qPCR) has been used for GPV detection; Woźniakowski et al. [9] established TaqMan qPCR for both classic GPV and MDPV that targeted the ITR region of the viruses. Confusion when calculating results may occur because the genomes of GPVs and MDPVs share two ITR repeat regions. Additionally, mutations and deletions in the ITR repeat regions were found recently, which may cause false negative results [10–12]. Recently, novel GPVs (designated as N-GPVs) causing short beak and dwarfism syndrome (SBDS) in Cherry Valley ducks, Pekin ducks and Mule ducks were found in China [13–15]. Niu X et al. [16] and Wang J et al. [17] proposed a TaqMan-based real-time PCR method for the specific detection of N-GPV. The VP3 gene of N-GPV was chosen as the target gene for primer-pairs and probe design, but this only detected N-GPV, not classic GPV (cGPV). Here, we report on the development of a specific TaqMan qPCR for both cGPVs and N-GPVs, which targets the NS differences between GPVs (including cGPVs and N-GPVs) and MDPVs. Based on the host specificity of cGPV (geese, Muscovy ducks, swans and *Anser cygnoides*) and N-GPV (Cherry Valley ducks, Peking ducks and Mule ducks), our TaqMan qPCR can be used for the specific differentiation of cGPV and N-GPV, coupled with host specificity.

## Results

### Primers and probe analysis

A total of 52 NS gene sequences (37 GPV strains and 15 MDPV strains) were retrieved from the GenBank database. For the forward primer GPV-qF (5′- TAGGGAGGAGTTAGAAGA-3′) (position 1554–1571), 36 of 37 (97.30%) matched the designed forward primer and only 1 (strain SDLY1602, GenBank accession number MF441222) of 37 had a mismatched sequence. For the reverse primer GPV-qR (5′-TACTTATGACAATTCTATGGATG-3′) (position 1689–1711), 36 of 37 (97.30%) matched the designed reverse primer and only 1 (strain GPV GER, GenBank accession number KU684472) of 37 indicated a sequence mismatch. For the probe GPV-qP (5′-AGAGAAGCA**R**GAACAATTACCAGGT-3′) (position 1649–1673), 21 of 37 (53.76%) shared the “AGAGAAGCAGGAACAATTACCAGGT” sequence and 12 of 37 (32.43%) shared the “AGAGAAGCAAGAACAATTACCAGGT” sequence. Thus, the probe (FAM-5′- ACCTGGTAATTGTTCYTGCTTCTCT-3′-Eclipse) was designed with a degenerate base (C/T = Y), which allowed the designed GPV-qP probe to cover 33 of 37 (89.19%) isolates. When the 17 MDPV isolates were compared at position 1649–1673, 8 of 15 (53.33%) shared AGAAAACCCGTGGGGACTATCAGGT, 4 of 15 (26.67%) shared AGAAAACCCGT**C**GGGACTATCAGGT, 2 of 15 (13.33%) shared AGAAAACCCGTGGGGAGTATCAGGT and 1 of 15 (6.67%) shared AGAAAACTCGT**G**GGGACTATCAGGT. These data showed significant differences between GPVs and MDPVs within the probe design region. The primers GPV-qF and GPV-qR and the TaqMan probe GPV-qP variations are listed in Table 1.

**Table 1:**
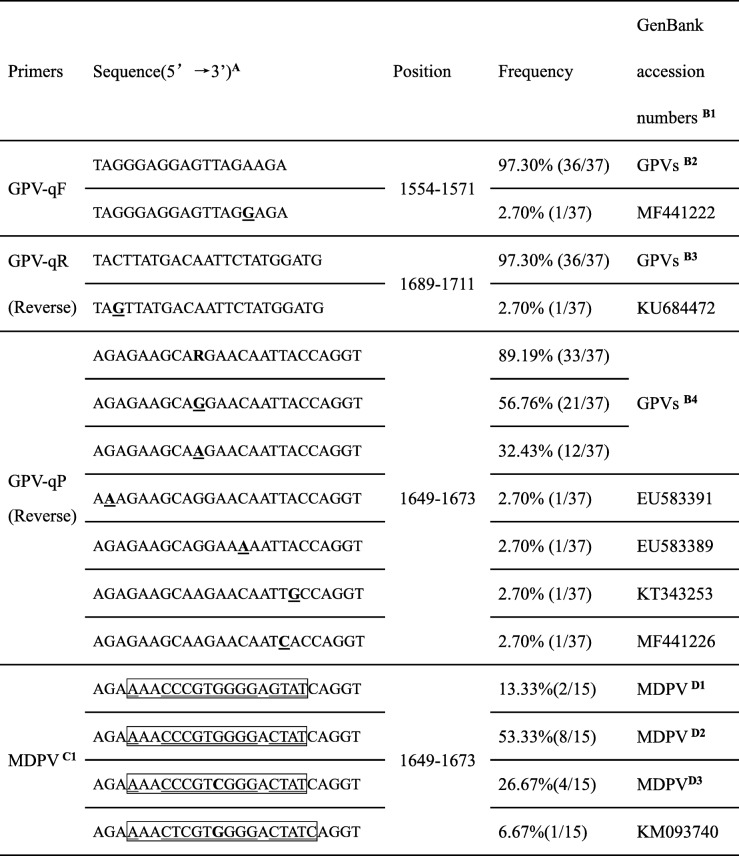
Sequence variation in multiple sequences alignment between GPVs and MDPVs

^A^The variation marks as Bold and underline

^B1^The GenBank accession numbers of GPVs (including N-GPVs) strains used in this study as following: KC996729, KT598506, HQ891825, KT598505, JF333590, KY511292, KR136258, U25749, KC996730, KC478066, KY475562, EU583390, KM272560, KC184133, EU583392, KR091960, EF515837, KC178571, AF416726, KU684472, KR091959, EU583391, EU583389, KT935536, KT935531, KX384726, KY679174, KT751090, MF441225, MF441224, MF441227, MF441223, MF441221, MF441222, MF441226, KU844283 and KT343253. Only mark the variation GenBank accession numbers of GPV (including N-GPV) strains

^B2^The GenBank accession numbers of GPVs exclude MF441222

^B3^The GenBank accession numbers of GPVs exclude KU684472

^B4^The GenBank accession numbers of GPVs exclude EU583391, EU583389, KT343253 and MF441226

^**C1**^ MDPV sequence compared with the GPV-qP, the variations are mark with square box, the results showed that the designed GPV-qP is specificity

MDPV ^**D1**^ The GenBank accession numbers of MDPV: U22967 and X75093

MDPV^**D2**^The GenBank accession numbers of MDPV: KU844282, KU844281, KT865605, KX000918, JF926697, KC171936, JF926698 and KY744743

MDPV^**D3**^The GenBank accession numbers of MDPV: KY069274, JF926695, KY511293 and JF926696

### Real-time PCR

**Fig. 1:**
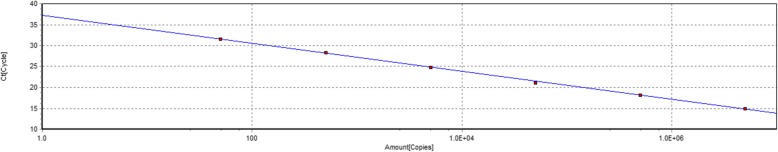
Standard curve of TaqMan qPCR assay

**Fig. 2:**
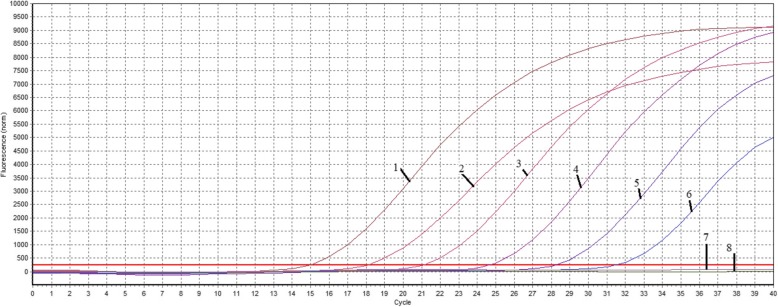
Sensitivity test of TaqMan qPCR assay. 1–7: a serial of ten-fold dilutions plasmid DNA (5.02 × 10^6^ to 5.02× 10^0^ copies/μl); 8: negative control (Nuclease-free water)

**Fig. 3:**
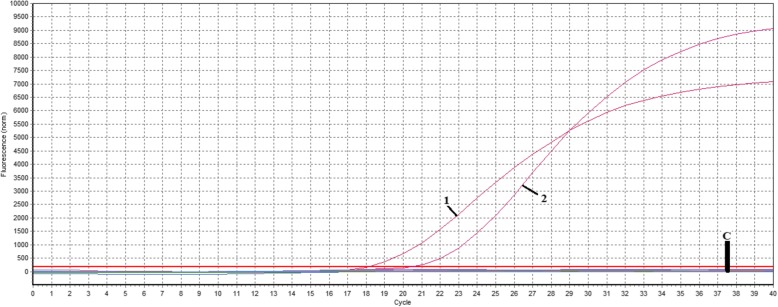
Specificity test of TaqMan qPCR assay. 1: cGPV; 2: N-GPV; Controls: *E. coli., P.M., R.A.*, S.S., MDPV, N-MDPV, DAdV-A, DEV, GHPV, DHAV-1, DHAV-3, ATmV, AIV, MDRV, and N-DRV. No positive fluorescence signal occurred with these pathogens

The CalQplex software (Mastercycler ep realplex, Eppendorf, Germany) automatically uses the Ct values from plasmid pT-G serial dilutions to calculate the standard curve of the TaqMan real-time PCR assay. The results show Ct values as a function of the amount of different copies of DNA. The standard curve of the assay showed linearity with a slope of − 3.344, Y-intercept of 37.19, efficiency of 99% and R2 of 0.999 (Fig. 1). The detection limit was assessed at 5.02 ×  10^1^ copies/μl (Fig. 2). For the specificity analysis, both cGPV and N-GPV produced strong fluorescent signals. No cross-reactivity was detected with other pathogens (i.e., *E. coli., P.M., R.A.*, S.S., MDPV, N-MDPV, DAdV-A, DEV, GHPV]) or cDNA (i.e., DHAV-1, DHAV-3, ATmV, AIV, MDRV and N-DRV) (Fig. 3). For intra- assay variability, low SD values (ranging from 0.11 to 0.55) were observed for each dilution mean and the CVs ranged from 0.58 to 1.74%; for inter-assay variability, low SD values (ranging from 0.12 to 0.75) were observed for each dilution mean and the CVs ranged from 0.66 to 2.37% (listed in Table 2).

**Table 2:** Intra- and inter-assay reproducibility for TaqMan qPCR

Concentration of standard plasmid (copies/μl)	Intra-assay variability		Inter-assay variability	
	$\overline{X}\pm SD$	CV (%)	$\overline{X}\pm SD$	CV (%)
5.02 × 10^5^	18.17 ± 0.11	0.58	18.19 ± 0.12	0.66
5.02 × 10^3^	24.56 ± 0.19	0.78	24.69 ± 0.22	0.90
5.02 × 10^1^	31.55 ± 0.55	1.74	31.71 ± 0.75	2.37

### Clinical samples application

TaqMan qPCR and cPCR were simultaneously performed on clinical samples. The results are summarized in Table 3. The frequency of GPVs (including cGPVs and N-GPVs) was determined to be 37 and 32% by TaqMan qPCR and cPCR, respectively. As summarized in Table 4, for 25 Mule duck embryos, 3 embryos (12%) and 2 embryos (8%) tested positive using the TaqMan qPCR and cPCR methods, respectively. For newly hatched Mule ducklings, 5 ducklings (20%) and 3 embryos (12%) tested positive using the TaqMan qPCR and cPCR methods, respectively. Moreover, all cPCR samples tested positive when using the TaqMan qPCR.

**Table 3:** Detection results in the clinical samples by TaqMan qPCR and conventional PCR

Birds	Number	qPCR		cPCR		Results^a^
		Positive number	Ratio (%)	Positive number	Ratio (%)	
geese	25	11	44	9	36	Classic GPV
Muscovy ducks	25	3	12	3	12	Classic GPV
Cheery Valley ducks	25	14	56	13	52	N-GPV
Mule ducks	25	9	36	7	28	N-GPV
Total	100	37	37	32	32	/

^a^ Results means the detection results of GPV or N-GPV infection in the clinical samples based on the host

**Table 4:** Detection results of vertical transmission in Mule ducks

Species		Number	Positive		Copy number for positive samples (copies/μl)	
			qPCR	cPCR	Both ^a^	Only ^b^
Mule ducks	embryos	25	3	2	3.27 × 10^3^, 9.82 × 10^2^	6.17× 10^1^
	ducklings	25	5	3	1.75 × 10^3^, 7.29 × 10^2^, 5.48 × 10^3^,	1.09 × 10^2^, 2.43 × 10^2^

^a^ Both means the samples tested with both qPCR and cPCR positive

^b^ Only means the samples only tested with qPCR positive

A total of 12 (two of each cPCR-positive samples were chosen randomly from different origins, i.e., geese, Muscovy ducks, Cherry Valley ducks, Mule ducks, embryos and ducklings) cPCR-positive amplicons were harvested, purified, T-A cloned and sequenced in both directions at Sangon (Shanghai, China). All 12 cloned sequences shared 100% matched with the primers (GPV-qF and GPV-qF), 8 of 12 N-GPV-positive samples (66.67%) shared the GPV-qP probe sequence “AGAGAAGCAGGAACAATTACCAGGT”, and 4 of 12 classic GPV-positive samples (33.33%) shared the sequence “AGAGAAGCA**A**GAACAATTACCAGGT”.

## Discussion

Real-time PCR technology has proven beneficial for studying the role of viral reactivation, which can help clarify the progression of disease. In contrast to conventional PCR, fluorescence intensity during each PCR cycle is used to quantify real-time PCR amplified products. Currently, there are two major types of real-time PCRs based on fluorescent dye and specificity: double-stranded DNA-intercalating dye (e.g., SYBR Green I, Eva Green) and HybProbe-based real-time PCR (e.g., TaqMan-probe, MGB-probe). TaqMan-probe is a representative of the hydrolysis type and is designed to bind to a specific site of the target DNA; this probe has shown improved specificity in distinguishing between closely related strains [6–8].

In this study, the real-time PCR probe we designed indicated that 21 of 37 sequences (53.76%) shared the “AGAGAAGCAGGAACAATTACCAGGT” sequences. These 21 sequences all belonged to the classic-GPV group. Twelve of thirty-seven sequences (32.43%) shared the “AGAGAAGCAGGAACAATTACCAGGT” secxquence. These 12 sequences all belonged to the N-GPV group. We designed two probes, one (designated as GPV-qP0) was synthesized with “ACCTGGTAATTGTTCCTGCTTCTCT” and the other (designated as GPV-qP) was synthesized with “ACCTGGTAATTGTTCYTGCTTCTCT” using a degenerate base (C/T = Y). After optimizing the real-time PCR, both probes could be used for the quantification of classic GPV and N-GPV, sharing the same detection limit of 5.02 × 10^1^ copies/μl. To cover the most frequently occurring GPV, the GPV-qP was then chosen as the TaqMan probe for the present research.

In this study, a total of 52 NS gene sequences (37 GPVs and 15 MDPVs) were compared for primer and probe design. Previous studies showed that NS genes shared characteristic variations between GPVs and MDPVs that could be used to design more precise primers and probes [18, 19]. Using a similar strategy, a TaqMan real-time PCR for the detection and quantification of GPV was developed and evaluated. This TaqMan real-time PCR cannot distinguish between cGPV and N-GPV. However, based on the epidemiological status and characteristics of cGPV and N-GPV our method can be used for differentiating between cGPV and N-GPV based on host specificity. Positive fluorescence from goslings and Muscovy ducklings was considered cGPV positive, while positive fluorescence from Cherry Valley ducks, Pekin ducks and Mule ducks was considered N-GPV positive.

Previous studies provided evidence that cGPV could spread via vertical transmission in geese [20, 21]. Similarly, there was possible vertical transmission of N-GPV between breeder Cherry Valley and Pekin ducks to their ducklings [22, 23]. Classic MDPV shared the same phenomenon of possible vertical transmission, similar to our recent work [18]. In this study, we demonstrated that N-GPV appeared to possible vertically transfer from breeder Mule ducks to ducklings. Thus, future country-wide surveillance in Mule ducks should be enhanced.

## Conclusions

Based on the characteristic variable regions of NS genes in GPVs and MDPVs, we developed a specific detection of cGPV and N-GPV by TaqMan real-time PCR assay. Moreover, cGPV and N-GPV could be distinguished using the assay coupled with host specificity. Furthermore, our results demonstrated that N-GPV may be able to transmit vertically from breeding Mule ducks to ducklings.

## Methods

### Primers and probe selection

Previous studies demonstrated that the NS gene homology between GPVs (cGPVs and N-GPVs) and MDPVs ranged from 80.8 to 83.4% and can be used for GPVs and MDPVs differentiation [18, 19]. After a bioinformatics analysis of the NS genes of GPVs (cGPVs and N-GPVs) and MDPVs specific primers and a probe were designed using Primer Premier Software version 5.0 (Premier Biosoft, Palo Alto, CA, USA) following a similar strategy that we used to develop a specific TaqMan-based real-time PCR for MDPV. Detailed information regarding the primers and probe is shown in Table 1. The amplicon was 158-bp in length. The GPV-qF (5′- TAGGGAGGAGTTAGAAGA-3′), the GPV-qR (5′- CATCCATAGAATTGTCATAAGTA-3′), and the GPV-qP (FAM-5′- ACCTGGTAATTGTTCYTGCTTCTCT-3′-Eclipse) were synthesized by a commercial company (TaKaRa, Dalian, China).

### Bacteria DNAs, viral DNAs and cDNAs preparation

Bacterial genomic DNA [i.e., *Escherichia coli* (*E. coli.*), *Pasteurella multocida* (*P.M.), Rimerella anatipstifer* (*R.A.*) and *Salmonella* spp. (S.S.)] were extracted using EasyPure Bacteria Genomic DNA Kit (TransGen Biotech, Beijing, China).

Viral DNA [i.e., cGPV, N-GPV, MDPV, novel recombinant Muscovy duck parvovirus (N-MDPV), Duck adenovirus A (DAdV-A), duck enteritis virus (DEV), duck origin-goose haemorrhagic polyomavirus (GHPV)] and viral RNA [i.e., duck hepatitis virus type 1 and 3 (DHAV-1 and DHAV-3), Avian Tembusu virus (ATmV), H9N2 subtype avian influenza virus (AIV), Muscovy duck reovirus (MDRV) and novel duck reovirus (N-DRV)] were extracted using EasyPure Viral DNA/RNA Kit (TransGen Biotech, Beijing, China).

The cDNA of RNA viruses (DHAV-1, DHAV-3, ATmV, AIV, MDRV and N-DRV) was prepared with isolated RNA (approximately 100 ng for each) using TransScript II One-Step gDNA Removal and cDNA Synthesis SuperMix (TransGen Biotech, Beijing, China).

DNA and cDNA were quantified using a NANODROP 2000 spectrophotometer (Thermo Scientific, Waltham, MA, USA) and stored at − 80 °C until use.

### Plasmid construction

The partial NS gene of cGPV (strain G7, GenBank accession number KR029617) [3] was amplified by PCR with the primer sets forward primer (GNSF) 5′-ATACATATTGCACTACCTGATAC-3′ and reverse primer (GNSR) 5′-TTATTGTTCATTTTCAGCATCATC-3′. The amplified PCR products were then analysed with electrophoresis on 1.0% agarose gels. The expected PCR amplicons were T-A cloned using the pMD18-T Vector Cloning Kit (TaKaRa, Dalian, China). The recombinant plasmids were then sequenced in both directions using the Sanger method by a commercial company (Sangon, Shanghai, China). The selected plasmid, pT-G, was quantified using a NANODROP 2000 spectrophotometer (Thermo Scientific, Waltham, MA, USA). The number of plasmid pT-G copies was calculated using the following formula [24]. Ten-fold dilutions of the plasmid pT-G, ranging from 5.02 × 10^7^ to 5.02 × 10^0^ copies/μl, were prepared with EASY Dilution (TaKaRa, Dalian, China). Each diluted plasmid, with different aliquots, was stored at − 80 °C until use.

### Real-time PCR protocol optimization

The TaqMan qPCR assay was developed and validated with a Mastercycler ep realplex (Eppendorf, Germany). Different concentrations of the primers and probe were prepared into reaction tubes to optimize the assay by evaluating the highest fluorescence and lowest threshold cycle (Ct). The reaction concentrations were determined as follows: 12.5 μl of Premix Ex Taq (Probe qPCR, TaKaRa, Dalian, China), 0.6 μl of each primer (GPV-qF and GPV-qR, 10 μmol/l each), 1.2 μl of probe (GPV-qP, 10 μmol/l), 1 μl of DNA template, and Nuclease-free water in an amount to adjust the total reaction volume to 25 μl. The following thermoprofile was set: 1 cycle of 95 °C for 30 s, 40 cycles of 95 °C for 5 s, 58 °C for 10 s, and 72 °C for 15 s.

### Analytical sensitivity, specificity and reproducibility

Ten-fold dilutions of plasmid pT-G, ranging from 5.02 × 10^6^ to 5.02 × 10^0^ copies/μl, were then used to determine the sensitivity. Ten ng of DNA (i.e., *E. coli.*, *P.M., R.A.,* S.S., cGPV, N-GPV, MDPV, N-MDPV, DAdV-A, DEV, GHPV]) or cDNA (i.e., DHAV-1, DHAV-3, ATmV, AIV, MDRV and N-DRV) were used for the specificity analysis. All of the reactions were conducted in triplicate simultaneously.

To determine the reproducibility of the real-time PCR, plasmid pT-G at concentrations of 5.02 × 10^5^, 5.02 × 10^3^, and 5.02 × 10^1^ copies/μl were used to evaluate the coefficient of variation (CV). These plasmids were repeatedly amplified three different times daily to assess intra-assay variability and three different times weekly to assess inter-assay variability. The CVs were calculated according to the formula using the geometric mean Ct value deviation.

### Clinical samples application

A total of 100 individual dead suspected cases of infected waterfowls (geese, Muscovy ducks, Cherry Valley ducks, and Mule ducks, 25 birds in each group on the basis of species) (Table 3) were used to validate the TaqMan qPCR assay. These birds were collected from privately owned animals via participating veterinary hospital (namely as Poultry Disease Treatment Centre, a department of our institute). Each liver tissue was centrifuged at 4000 rpm at 4 °C for 30 min after mechanical grinding. Viral DNA was extracted with EasyPure Viral DNA/RNA Kit (TransGen Biotech, Beijing, China). Conventional PCR (cPCR) was also performed simultaneously to detect infections in the above samples [25]. Based on the host specificity of cGPV and N-GPV, positive signals from geese and Muscovy ducks were considered to be cGPV-positive, whereas positive signals from Cherry Valley ducks and Mule ducks were considered to be N-GPV-positive.

### Vertical transmission application

Previous studies confirmed that cGPV could spread through vertical transmission to susceptible young goslings via eggs. The same phenomenon can also be found with N-GPV in Cherry Valley ducks and Pekin ducks. To test the hypothesis that N-GPV could be vertically transmitted in Mule ducks, 25 Mule duck embryos (18-day post fertilization) and 25 newly hatched Mule ducklings (1-day-old), were collected from farms where N-GPV infections has previously occurred. The liver of each embryo and newly hatched duckling was designated as one sample. Viral DNA was extracted with EasyPure Viral DNA/RNA Kit (TransGen Biotech, Beijing, China). These samples were also simultaneously assayed using the cPCR method.

## Data Availability

All datasets are available from the corresponding author on reasonable request.

## Abbreviations

AIV: Avian influenza virus
ATmV: Avian Tembusu virus
cGPV: Classic goose parvovirus
cPCR: Conventional PCR
Ct: Threshold cycle
CV: Coefficient of variation
DAdV-A: Duck adenovirus A
DEV: Duck enteritis virus
DHAV-1 and DHAV-3: Duck hepatitis virus type 1 and 3
*E. coli.*: *Escherichia coli*
GHPV: Duck origin-goose haemorrhagic polyomavirus
GPV: Goose parvovirus
ICTV: International Committee on Taxonomy of Viruses
ITR: Inverted terminal repeats
MDPV: Muscovy duck parvovirus
MDRV: Muscovy duck reovirus
N-DRV: Novel duck reovirus
N-GPV: Novel GPV
N-MDPV: Novel recombinant Muscovy duck parvovirus
NS: Nonstructural
ORF: Open reading frame
*P.M.*: Pasteurellamultocida
*R.A.*: Rimerella anatipstifer
R^2^: Correlation
*S.S.*: Salmonella spp.
SBDS: Short beak and dwarfism syndrome
TaqMan qPCR: TaqMan real-time PCR
